# Proofs for PLOS ONE Paper : Does Pharmaceutical Pricing Transparency Matter? Examining Brazil’s Public Procurement System

**DOI:** 10.1186/s12992-015-0118-8

**Published:** 2015-08-04

**Authors:** Jillian Clare Kohler, Nicholas Mitsakakis, Faridah Saadat, Danalyn Byng, Martha Gabriela Martinez

**Affiliations:** Leslie Dan Faculty of Pharmacy, University of Toronto, 144 College St., Toronto, ON M5S 3M2 Canada; Munk School of Global Affairs, University of Toronto, 1 Devonshire Place, Toronto, ON M5S 3K7 Canada; Toronto Health Economics and Technology Assessment (THETA) Collaborative, 144 College St., Toronto, ON M5S 3M2 Canada; Institute for Health Policy, Management and Evaluation, University of Toronto, 155 College St., Toronto, ON M5T 3M6 Canada

**Keywords:** Pharmaceutical procurement, Brazil, Banco de Preços em Saúde, Drug prices, Transparency, Transparency tool, Medicine prices

## Abstract

**Background:**

We review procurement and pricing transparency practices for pharmaceutical products. We specifically focus on Brazil and examine its approach to increasing pricing transparency, with the aim of determining the level of effectiveness in lower prices using a tool (*Banco de Preços em Saúde*, BPS) that only reveals purchase prices as compared to other tools (in other countries) that establish a greater degree of price transparency.

**Methods:**

A general report of *Preços em Saúde* (BPS) and *Sistema Integrado de Administração de Serviços Gerais* (SIASG) pricing data was created for 25 drugs that met specific criteria. To explore the linear time trend of each of the drugs, separate regression models were fitted for each drug, resulting in a total of 19 models. Each model controlled for the state variable and the interaction between state and time, in order to accommodate expected heterogeneity in the data. Additionally, the models controlled for procurement quantities and the effect they have on the unit price. Secondary analysis using mixed effects models was also carried out to account for the impact that institutions and suppliers may have upon the unit price. Adjusting for these predictor variables (procurement quantities, supplier, purchasing institution) was important to determine the sole effect that time has had on unit prices. A total of 2 x 19 = 38 models were estimated to explore the overall effect of time on changes in unit price. All statistical analyses were performed using the R statistical software, while the linear mixed effects models were fitted using the lme4 R package.

**Results:**

The findings from our analysis suggest that there is no pattern of consistent price decreases within the two Brazilian states during the five-year period for which the prices were analyzed.

**Conclusions:**

While the BPS does allow for an increase in transparency and information on drug purchase prices in Brazil, it has not shown to lead to consistent reductions in drug purchase prices for some of the most widely used medicines. This is indicative of a limited model for addressing the challenges in pharmaceutical procurement and puts into question the value of tools used globally to improve transparency in pharmaceutical pricing.

**Electronic supplementary material:**

The online version of this article (doi:10.1186/s12992-015-0118-8) contains supplementary material, which is available to authorized users.

## Background

Good governance is recognized as an instrumental component of healthy health systems [1, 2]. Improving good governance can ideally reduce the likelihood of corruption. We understand good governance as consensus- oriented, accountable, transparent, responsive, equitable and inclusive, effective and efficient, follows the rule of law and is participatory [3]. In theory, supporting good governance should make any system less vulnerable to corruption. Despite the growing recognition of the importance of good governance, corruption continues to be reported in national and global health systems across a range of environments and has undermined progress in health services delivery and system strengthening at a time when investments in global health interventions has never been higher [4, 5]. Health-related corruption occurs in both the private and public sphere in high-income countries, developed country settings, and in multilateral initiatives [4]. Procurement in pharmaceuticals is an area where corruption is always a threat [6]. This includes collusion in bidding, “fixed” procurement bidding and kickbacks to public officials in order to gain support for a bid [6]. In order to undercut the threat of corruption in procurement, particularly in pharmaceutical procurement practices, governments across the globe are turning to good governance measures such as drug pricing transparency. However, whether pricing transparency actually leads to positive outcomes, such as lower drug prices still remains.

Our paper thus examines whether in fact increased transparency in drug procurement prices actually results in any discernible difference in drug prices and with that, ideally, improving access to medicines. We focus our paper on Brazil and examine its approach to increasing pricing transparency. More specifically, we aim to examine if pricing transparency leads to lower drug prices over time. We justify our choice of Brazil’s federal public institutions and its suppliers because at the federal level, reporting procurement pricing information is mandatory. Thus, we were able to extract such drug pricing information from a governmental online database to conduct an analysis on the effect that pricing transparency has had on drug purchase prices in two Brazilian states over a five-year period. We set out to answer the following specific questions: What does the BPS database show with regards to drug prices over time? What does the database illuminate with regards to drug prices in two socioeconomically different Brazilian states? Is the BPS an effective transparency tool to decrease drug prices?

In recognition of the challenges that are present within pharmaceutical procurement due to the potential for pricing distortions, the analysis was structured to address and control for procurement quantities (economies of scale), and supplier and institution/purchaser (potential sources of corruption). As we noted above, drug procurement is often a common site for corruption, further exacerbating inequities in access to medicines amongst the population if, for example, drugs procured have prices that are out of reach. It is well known that when corruption is present, it diverts critical government resources from health services and products, which usually has the largest negative impact on the poor and marginalized. Each year, over US$ 4.1 trillion is spent worldwide on providing health services [7]; but a significant amount of this money is wasted due to corruption. In the area of procurement alone, it is estimated that between 10 % and 25 % of global spending on public procurement is lost to corruption [8].

The primary focus of our paper is to investigate the impact, if any, of pricing transparency on pharmaceutical price trends in two states in Brazil – Paraiba and São Paulo. These states have starkly different socioeconomic conditions, representing states on either end of the socioeconomic spectrum. As is noted later in the paper, the burden of spending on medicines is most significant for the poorest of Brazil’s population. We therefore sought to see if pricing transparency has had any impact on alleviating this and whether it helps establish equity among the population. In our analyses the state of Paraiba represents the poorest of Brazil’s population, and São Paulo the richest. We propose that Brazil’s approach to lowering drug prices through the BPS is limited. We hypothesize that it does not fully address all sources of possible corruption and inefficiency, nor does it provide enough incentive for pharmaceutical suppliers to compete with lower prices. Examples of alternative pricing procurement models are provided in multi-country case studies later in the report.

### Access to medicines and Brazil

In Brazil, access to health services and essential medicines has been a recognized constitutional right since 1988. The country’s publicly-funded universal health system, *Sistema Unico de Saude* (SUS) covers all members of the population. In addition to public hospitals and clinics, SUS oversees the distribution of publicly funded medicines that are listed on Brazil’s Essential Medicines List (EML), as well as medicines for rare diseases and those affecting small groups (e.g. anti-retroviral drugs for HIV/AIDS, Hepatitis B and C) [9]. Very few of Brazil’s private health plans offer medication coverage, so there are many privately insured people who still rely on SUS for medicines [10].

While SUS is committed to ensuring equitable access to medicines, disparities still exist. The burden of spending on medicines, not surprisingly, is most significant for the poorest of Brazil’s population. Households belonging to the lowest income quintile still pay out-of-pocket for nearly one quarter of their drug purchases [11]. In 2008–2009, the proportion of out-of-pocket spending on medications in relation to family income was highest for families in the bottom decile – approximately three times higher than those in the top decile [12].

Demographic changes as well as persistent inefficiencies throughout the pharmaceutical supply chain have been major burdens on the government and have had significant impacts on the availability and accessibility of drugs for the country’s population [10, 11]. In addition to out-of-pocket spending burdens, 40 % of medicines that are prescribed in public health care settings are not readily available in pharmacies due to low resources and high costs [10, 11].

Pharmaceuticals typically account for about 10 to 30 % of health budgets in low-income countries [13], so the appeal of finding effective ways to push down prices is obvious. A well-tried approach is to improve transparency in the pricing of pharmaceuticals – a practice that is gaining traction globally as a method to help reduce pharmaceutical expenditures. In recent years, global initiatives such as the Medicines Transparency Alliance (MeTA) and international organizations such as the World Health Organization have led efforts to make information on price, quality, availability and promotion of medicines publicly available [14]. As a result, there has been a growth in the creation of databases that make purchasing information of medicines and other medical products publicly available on the Internet. Such public intelligence can, ideally, reveal any potential areas of abuse in pricing, avoid price variations caused by inefficiencies, and eliminate price differences caused by asymmetrical information among medicine suppliers and purchasers [15].

### Pharmaceutical Procurement: creating a more competitive market

Most medicines are not sold in competitive commodity markets. The pharmaceutical market is oligopolistic with few competitors (producers and suppliers) who are able to drive up prices given the informational asymmetry that exists between themselves and their purchasers. Perfect competition does not exist when it is the producers and suppliers that have the greatest influence and market power to determine the prices of their products. The consumer typically has little leverage over driving prices down, particularly on products that are essential for health and well-being such as pharmaceuticals.

Still, as major purchasers of pharmaceuticals, governments have introduced public sector procurement as an opportunity to create increased competition among suppliers. An advantage of this is that it would allow a choice between multiple suppliers, who compete for lower prices. To increase competition among suppliers, numerous governments have also introduced web-based procurement portals with varying degrees of publicly available information. This approach to “pricing transparency” ranges from publically available information on suppliers who have won bids to procure products, to purchase price information, to complete disclosure of every stage of the procurement process (including revealing the competing bids and the prices that they were set at). Depending on the level of price transparency within these procurement systems, governments have realized different levels of cost savings.

There are many hypothesized benefits to increasing transparency in the purchasing of medicines and medical supplies. The increase in accurate information should allow policy makers and procurement managers to bid on fair prices, track prices over time, and more accurately estimate budget requirements [16]. Additionally, transparency in pricing can expose irregular price mark-ups, it can assist in the avoidance of drug shortages through accurately forecasting quantities of essential supplies, it can decrease risk of theft or spoilage of supplies, and the purchase of counterfeit drugs can be avoided [14]. Theoretically, increasing price transparency has the potential to increase accessibility to drugs – both in terms of decreased prices and greater availability. It can potentially act as a powerful incentive for producers and suppliers to raise the quality and lower the price of their goods. Ideally, it also helps reduce the likelihood of corruption, thus affecting drug prices.

### Brazil’s Approach to pricing transparency

In an effort to improve transparency and accountability in the pharmaceutical system, in 1998 Brazil’s Federal Government implemented, under the leadership of then Minister of Health, Jose Serra, the Banco de Preços em Saúde (BPS). The BPS was to be used as a transparency measure to facilitate the centralization of pricing information, and to decrease the high cost of medicines and medical supplies [17, 18].

The BPS, initially known as the Data Bank of Hospital Prices (Banco de Preços Praticados na Área Hospitalar, BPPH), began as an Ordinance that made it mandatory for all federally funded hospitals with 320 beds or more to publish purchasing prices of medical supplies on the Ministry website [19]. Today, it works as a free and open online information system that records, stores, and makes available the prices of medicines and health products purchased by public and private institutions registered in the system. The creation of the BPS is not unique; as noted throughout this paper later, similar online drug pricing portals are also available in the neighbouring Mercosur countries [20, 21].

The objectives of the BPS are as follows: (1) to manage market information of medicines and other health products, particularly information that relates to the supply and demand of such goods; (2) to monitor the price trends of prices of medicines and other health products; (3) to increase access to buyers for suppliers; (4) to guide public managers in decision making by allowing price comparisons; and lastly, (5) to increase transparency in the allocation of SUS resources by establishing social control through the introduction of open online access to the BPS [21]. The BPS discloses purchasing prices of medicines that are paid for by public institutions at the federal, state and municipal levels of government, as well as of private and international institutions that have been registered in the system (e.g. some NGOs, private clinics/hospitals) [22]. While private and public institutions provide purchasing information voluntarily, federal public institutions are required to do so by law.

The BPS registry is publicly available online without restrictions. The price bank generates excel reports with the following variable headings: date of purchase; quantity; unit price; per cent comparative to the lowest price; brand; form of bidding; the country of origin of purchase; purchase receipt number; number of bids; vendor name; and manufacturers’ name and country. This information is readily available for a wide range of medical products [22]. As of 2012, there were 948 registered institutions, with a total of 15,180 purchases corresponding to 43,677 items with a total of R18.2 billion [23].

### Why look at São Paulo and Paraiba?

We sought to explore whether a transparency tool such as the BPS would result in different price outcomes in two states with very different socioeconomic conditions. The rationale behind this came from our understanding of the large negative impact that high drug prices has on the poor and marginalized. As is noted, the burden of spending on medicines is most significant for the poorest of Brazil’s population. We thought it necessary to analyze pharmaceutical pricing changes resulting from the BPS, a tool theorized to address inefficiencies within procurement, in two states – Paraiba and São Paulo. These states have starkly different socioeconomic conditions, representing states on either end of the socioeconomic spectrum. We therefore sought to see if the BPS has had any impact on alleviating this and would contribute to establishing equity among the population. In our analyses the state of Paraiba represents the poorest of Brazil’s population, and São Paulo the richest. Paraiba has an average nominal monthly income per capita of R$565 with 27.99 % of Paraiba’s households earning a nominal monthly income equal to or less than the state minimum wage [24]. São Paulo on the other hand has an average nominal monthly income per capita that is more than double that of Paraiba at R$1259 [21], with only 6.36 % of São Paulo’s households earning a nominal monthly income equal to or less than the state minimum wage [24]. Moreover, in the state of Paraiba, the poorest families (average monthly income of up to US$415) spend a monthly average of 4.0 % (equivalent to US$12.96) of their income on medicines, while the richest families (with an average monthly income of more than US$5,187) spend a monthly average of 1.7 % (equivalent to US$97.54) of their income on medicines. In the state of São Paulo, the poorest families (average monthly income of up to US$415) spend a monthly average of 5.7 % (equivalent to US$23.74) of their income on medicines, while the richest families (average monthly income of more than US$5,187) spend a monthly average of 2.9 % (equivalent to US$187.08) of their income on medicines. With such large differences between these states, we were keen to examine if pricing transparency matters across economic divides.

Several quantitative and qualitative studies have illustrated how the burden of corruption impacts the poor more heavily, given their limited ability to pay, resulting in limited access to the health care system [25]. Certainly within Brazil, it has been made clear that there is a discrepancy in proportion of out-of-pocket spending between rich and poor families within both states, this may be a simply inefficiencies in the system or possibly corruption. Without adequate tools that help ensure good governance within pharmaceutical systems, any funding allocated to treat health conditions may simply be wasted and inequality between rich and poor in access to health and pharmaceutical products will be aggravated [26]. For these reasons, it is important to determine whether the BPS is an adequate tool for decreasing drug prices.

### Alternative pharmaceutical procurement practices

To provide examples of alternative pharmaceutical practices and tools, we highlight examples from other Latin American countries. In the early 2000s, MERCOSUR Member States (Brazil, Argentina, Uruguay, Paraguay) and its associated countries, Bolivia and Chile, developed and approved an Agreement entitled the “Drug Policy of MERCOSUR, Bolivia and Chile”. The purpose of the Policy is to seek to improve state action, particularly with regard to four themes identified as key objectives for the countries of the region in the area of medicines:

- Expanding people’s access to drugs, considering the needs of different social groups;
- Ensuring the quality, safety and efficacy of the drugs circulating in the region;
- Promoting a culture of rational use of medicines;
- Creating an environment of research and development that supports better integration of the countries in the technology sector. [27]

Since then, efforts to increase access to medicines through the public sector have been seen across these countries. Initiatives in Argentina, UCAMAE in Uruguay, and in Chile show how governments are utilizing procurement systems and economies of scale to bargain and negotiate drug purchases in a centralized fashion. These efforts stem from a need to contain costs and ensure broad access to essential medicines. A key aspect to these programmes is the implementation of various pricing transparency approaches within their procurement systems. The following section will provide a review of these programmes, with a particular focus on the ways in which they vary in terms of intensity and scope of pricing transparency.

### Argentina

In 2001, Schargrodsky et al. analyzed the effects of the mandatory report of purchasing prices paid for medicines and medical supplies by 33 hospitals in Buenos Aires from August 1996 to December 1997. The government compiled the reported prices into a report, which was distributed back to the hospitals beginning in October of 1996. The study found that drug prices significantly decreased a month after the mandatory purchase price policy was implemented, but the trend was not continued and prices eventually began to increase over time [15]. The decrease in drug prices observed by Schargrodsky *et al*. took place before the report on drug prices was actually disseminated back to the hospitals. This suggests that the observed decreases in drug prices were caused by the anticipation of policy mandating the report of purchase prices, as well as the “fear” of possible chances of detection and punishment if prices were well beyond the average. Savedoff argues that merely publishing price information was not enough to improve efficiency [28]. In the absence of consequences procurement officers became accustomed to the process of reporting and the lack of further investigation into any poor performance.

In the midst of the economic crisis of 2001–2002, the government of Argentina decided to implement the national REMEDIAR program with the aim of providing free medicines to in-patients, low-income patients, children under 5 years of age, pregnant women, and elderly adults. Today, this program effectively provides coverage for all medicines on the Essential Medicines List for 16 million people across the country [29]. In addition, Argentina’s public sector procurement also consists of provincial and municipal programs. Although Argentina has no legal or regulatory provisions that dictate the pricing of medicines, the Ministry of Health produces a monthly Average Drug Price Index (IPPM) as well as an index of weighted prices based on the REMEDIAR Program [30]. Public sector tender bids recently became publicly available, with the REMEDIAR program updating its webpage to publicly publish bids and awards [31]. This move to increase the scope of pricing transparency can certainly be attributed to the country’s past lessons in the inefficiencies of merely publishing purchase prices to help ensure competitive drug prices, as highlighted in Schargrodsky et al’s 2001 study [15].

### Bolivia

Despite the existence of a National Essential Medicines Program (Programa de Medicamentos Esenciales de Bolivia, PNMEBOL), the Bolivian Government estimates that nearly 50 % of the population still experiences significant economic, cultural, and geographic barriers to accessing drugs [32]. Corruption in the pharmaceuticals chain has undercut much of the government’s efforts to improve access to medicines. Ongoing drug smuggling activity as well as counterfeit and adulterated drugs entering the market have undermined the aims of the PNMEBOL [32]. In recognition of these issues, Bolivia recently instituted a National Drug Policy. The policy rests on two main pillars: el *Sistema Nacional de Vigilancia y Control de Medicamentos* (National System for Monitoring and Medicine Control), and *Sistema Nacional Unico de Suministro* (Unified National Supply System). The National System for Monitoring and Control of Drugs is upheld by the country’s Medicines Act and its accompanying regulations. It allows the government to regulate the importing, production, distribution, sale, advertisement, and surveillance of drugs in order to protect patients against the potential hazards of fraudulent activity in the pharmaceutical supply and distribution chain.

The National Drug Policy is also focused on strengthening the *Central de Abastecimiento y Suministros* (CEASS, Center of Procurement and Supply) in order to ensure the availability of essential medicines at reasonable costs for the country’s network of public health institutions. It has established standards for the qualification of suppliers, best practices for the procurement of drugs through state-run institutions, as well as technical and quality requirements for the various types of procurement contracts. Bolivia’s *Sistema de Contrataciones Estatales* also allows for a competitive acquisition strategy to procure essential medicines of high quality, safety and efficiency. Overall, Bolivia has taken the necessary steps to optimize the use of government financial resources for purchasing pharmaceuticals, while also keeping in high consideration the health needs of the population.

### Chile

Public procurement in Chile is comprehensively governed both by the 2003 Law on Procurement and complementary regulations that guide the public procurement process. The implementation of these governing tools led to the formation of the *Dirección de Compras y Contratación Pública* (Public Procurement and Contract Direction) and its accompanying internet portal ChileCompra in 2010 [33, 34]. This e-procurement system (www.mercadopublico.cl) allows procurement processes to be centralized and digitized, negotiating multi-year agreements with suppliers for select products [35, 36].

For the specific procurement of drugs and medical devices, CENABAST (*Central Nacional de Abastecimiento*) is the single buying agency for the Chilean Ministry of Health. CENABAST is responsible for the procurement and distribution of essential medicines for Chile’s hospitals. To procure generic drugs, CENABAST aggregates hospitals’ demand, and releases calls for proposals through the ChileCompra e-marketplace. Awarded products are set at a fixed price for 6 months, until the process is repeated again (twice yearly). Non-generic drugs and vaccines are purchased by the Ministry of Health through CENABAST, with the Ministry determining quantity and delivery structure. In 2010, purchases made through CENABAST made up 36.6 % of total drug purchases in Chile [37].

### Paraguay

In 2009 Paraguay’s Ministry of Health developed an Essential Drugs List and an accompanying integrated logistic system for medicines and supplies. *The Sistema de Información y Control de Inventario Automatizado* (SICIAP, Automated Information and Inventory Control System) allows health officials to track medicines through the country’s supply chain of warehouses, hospitals and clinics. It is estimated that the new system will save approximately US$6.2 million each year through its ability to oversee potential shortages, avoid waste, and control proper storage and use of medicines [38].

### Uruguay

In 2005, Uruguay implemented a National Integrated Health System, within which a centralized public procurement unit for the purchase of medicines (UCAMAE, http://uca.mef.gub.uy) was created [39]. Public hospital pharmacies make their purchases through UCAMAE. According to Lalanne (2004), 55 % of the purchases through UCAMAE were done on behalf of the public health system, 19 % were for public hospitals, 36 % for public health insurance entities, and 45 % for drug stores and other private distribution channels [40]. This represents a significant opportunity to centralize purchases and drive down public spending on medicines.

The prior examples illuminate how efforts to improve transparency in pricing and procurement are widespread throughout Latin America. It is therefore compelling to conduct an investigation of the impact of price transparency over time in order to help governments understand what transparency practices can ensure in terms of positive outcomes that ensure broader access to medicines for their populations.

## Methods

### Data acquisition

Access to the *Banco de Preços em Saúde* was obtained by registering to the BPS public database online (bps.saude.gov.br). Once access was acquired, a general report (*Relatório geral BPS e SIASG*) of BPS and SIASG data was created for 25 drugs. These 25 drugs were selected because they constitute widely utilized therapeutics on Brazil’s *Relação Nacional de Medicamentos Essenciais* (RENAME), the country’s national EML. These drugs are provided to citizens at no-cost through SUS [41], and are required to be available at all times in adequate amounts and dosages, with assured quality and adequate information, and at a price an individual and the health system can afford [42].

The reports were created by selecting *Equipamentos e artigos para uso medico, dentario* under *Grupo CATMAT*, *Drogas e medicamentos* under Classe CATMAT, the drug name under *Descrição Item* and the dates (dd/mm/yy) 01/01/2002 to 07/05/2013 under *Data de Compra*. Twenty-five general reports were created for 25 drugs. The 25 drugs were matched against specific criteria: a) data is available for an 8 year span (2005–2013), b) drug is listed on the 2012 RENAME, and c) drug is a widely utilized in its class of therapeutics. 6 drugs did not meet the criteria, and the 19 drugs that were chosen were as follows: acyclovir, amoxicillin, aspirin, atenolol, captopril, diazepam, dipyrone, enalapril, fluoxetine, furosemide, glibenclamide, hydrochlorothiazide, metformin, omeprazole, paracetamol, propanalol, ranitidine, tramadol, and warfarin (Table 1). Data from the general reports were extracted to show data (institution, date purchased, price paid ($R), quantity and supplier) from the states of São Paulo and Paraiba for each of the 19 drugs. The prices that are referred to are those at the procurement level between federal public institutions and the suppliers.

**Table 1:** The 19 medicines studied and analyzed from the Banco de Preços em Saúde

Drug	Dosage (mg)	Class
Aciclovir	200	Antiviral
Amoxicillin	500	Antibiotic
Aspirin	100	Antithrombotic agent
Atenolol	50	Antihypertensive (β-blocker)
Captopril	12.5	Antihypertensive (angiotensin-converting enzyme inhibitor)
Diazépam	5	Anxiolytic
Dipyrone	500	Analgesic
Enalapril	10	Antihypertensive (angiotensin-converting enzyme inhibitor)
Fluoxetin	20	Antidepressant
Furosemide	40	Diuretic
Glibenclamide	5	Antidiabetic
Hydrochlorothiazide	25	Diuretic
Metformin	850	Antidiabetic
Omeprazol	20	Drug for acid related disorder (proton pump inhibitor)
Paracétamol	500	Analgesic
Propranolol	40	Antihypertensive (βntihypert)
Ranitidine	150	Drug for acid related disorder (H2 receptor inhibitor)
Tramadol	50	Analgesic
Warfarin	5	Anticoagulant

### Statistical analysis

Data were described using means and standard deviations over all transactions, for each drug and state separately. To explore the linear time trend of each of the drugs that met the selection criteria, separate regression models were fitted for each drug, resulting in a total of 19 models. To accommodate for expected heterogeneity in the data, the models controlled for the state variable as well as the interaction between state and time. The models also controlled for procurement quantities and the effect they have on the unit price. Secondary analysis was also carried out to account for the impact that institutions and suppliers may have upon the unit price. Adjusting for these predictor variables (procurement quantities, supplier, purchasing institution) was important to determine the sole effect that time has had on unit prices. A total of 2 x 19 = 38 models were estimated to explore the overall effect of time on changes in unit price. All statistical analyses were performed using the R statistical software, while the linear mixed effects models were fitted using the lme4 R package [43].

### a) Primary analysis

For each pairing of drug and state, a linear regression model was estimated using the log of unit price as the dependent variable and time as the primary linear predictor (adjusted also for the log of the quantity within each transaction as unit prices appeared to be inversely proportional to purchase quantities in each transaction). The primary objective of this regression model was the investigation of any linear time trend of each drug’s unit price, therefore no other functional forms of the time variable were explored. Unit prices were also adjusted for inflation at each time point by dividing by a factor representing the inflation at the time point of the earliest transaction involving the specific drug. The inflation factor was calculated using the IPA-OG *Produtos Farmacêuticos* index (available from www.ipeadata.gov.br), which is a wholesale price index for pharmaceutical products. The index is available only up to the end of year 2007. We therefore needed to forecast its values for the remaining time horizon the drug transactions data covered (up the end of 2013). We used an ARIMA model for the forecasting selected based on the Akaike Information Criterion according to the auto.arima function in the forecast R package [44].

The quantity of drugs in each procurement is known to have a significant impact on the outcome variable (i.e. the effect of economies of scale), therefore the model controlled for this effect. If *up*_*ijk*_, *q*_*ijk*_ and *t*_*ijk*_, represent the unit price, quantity and time for transaction *i*, for supplier *j* and institution *k*, the model has the form:

$$ \log \left(u{p}_{ijk}\right)=a+{\beta}_1\times {t}_{ijk}+{\beta}_2\times log\left({q}_{ijk}\right)+{\beta}_3\times stat{e}_{ijk}+{\beta}_4\times \left( stat{e}_{ijk}\times {t}_{ijk}\right)+{\varepsilon}_{ijk}, $$

where the error *ε*_*ijk*_ is normally distributed with mean 0, and where *α*, *β*_*1*_*, β*_*2*_*, β*_*3*_*, β*_*4*_ represent the intercept and the coefficients for time, logged quantity, state and interaction between state and time respectively. Using Sao Paulo as the reference level for the state variable, the coefficient *β*_*1*_ represents the effect of time to the (deflated) logged unit price for the state of Sao Paolo, while the quantity *β*_*1*_ *+ β*_*4*_ represented the time effect for the Paraiba state. Equivalently, the quantity exp(*β*_*1*_) is the proportional change of the geometric mean of the deflated price unit over time for the state of Sao Paolo, while exp(*β*_*1*_ *+ β*_*4*_) represents the same time trend for the state of Paraiba. For either of these quantities values above 1 indicate an increasing trend over time, while decreasing trend corresponds to values below 1.

Non-stationarity problems were investigated with the use of the Augmented Dickey-Fuller test, using the adf.test function from the tseries R package [45].

### Secondary analysis

A secondary analysis of the data was carried out to account for potential clustering structures due to transactions involving the same supplier or the same institution. It was important to control for the effects of any given supplier and institution/purchaser as these may be sources of corruption within a procurement transaction. Linear mixed effects models were estimated, with the following form:

$$ \log \left(u{p}_{ijk}\right)={a}_{\mathit{\mathsf{1}}\mathit{\mathsf{j}}}+{a}_{\mathit{\mathsf{2}}\mathit{\mathsf{k}}}+{\beta}_1\times {t}_{ijk}+{\beta}_2\times log\left({q}_{ijk}\right) + {\beta}_3\times stat{e}_{ijk}+{\beta}_4\times \left( stat{e}_{ijk}\times {t}_{ijk}\right)+{\varepsilon}_{ijk}, $$

where the error *ε*_*ijk*_ and crossed random effects for supplier and institution *α*_*1j*_ and *α*_*2k*_ are all normally distributed and pairwise independent. Time trends are represented in a fashion similar to the primary analysis.

## Results

Table 2 shows the means and standard deviations of procurement quantities and (inflation adjusted) unit price over the number of transactions, for all 19 drugs and two states. Detailed descriptive statistics are included in the Additional files 1 and 2: Tables S1 and S2.

**Table 2:** Means and standard deviations for procurement quantity and deflated unit price for all drugs in Paraiba and Sao Paulo

Drugs	Paraiba			Sao Paulo		
	Number of transactions	Quantity	Unit price	Number of transactions	Quantity	Unit Price
warfarin 5	25	835.2 (837.92)	0.2548 (0.1825)	15	48842 (107277.37)	0.2724 (0.1639)
tramadol 50	36	3133.33 (4026.41)	0.5489 (0.7541)	19	8336.68 (29622.02)	0.9358 (1.2744)
ranitidine 150	41	7701.95 (8417.15)	0.1377 (0.3329)	64	1865.13 (9828.25)	0.6128 (1.4629)
propranolol 40	30	2369.8 (2309.97)	0.0630 (0.1674)	27	2018.81 (7632.9)	0.1070 (01042)
paracetamol 500	53	3450.43 (6303.88)	3.0024 (7.8183)	117	10697.24 (74257.98)	7.6817 (26.9272)
omeprazol 20	22	4316.91 (6573.12)	0.5314 (0.9903)	89	20094.3 (123487.28)	0.6563 (1.4910)
metformin 850	24	1180 (1012.03)	0.0887 (0.1116)	13	139276.92 (498986.65)	0.1061 (0.0802)
hydrochlorothiazide 25	18	2711.11 (2079.56)	0.2047 (0.7577)	12	669.25 (1684.59)	0.69833 (2.1274)
glibenclamide 5	27	1038.89 (805.28)	0.0425 (0.0631)	14	434.29 (521.31)	0.1056 (0.1267)
furosemide 40	48	3348.79 (5264.95)	0.6770 (1.3493)	36	1360.58 (5966.58)	0.5028 (0.6276)
fluoxetin 20	18	1316.67 (1585.15)	0.4489 (1.0811)	18	31890 (117633.3)	0.2202 (0.2812)
enalapril 10	22	1956.82 (3451.2)	0.0822 (0.1673)	20	905 (722.73)	0.9847 (2.5833)
dipyrone 500	55	1799.49 (3501.41)	0.5588 (1.8323)	214	4449.22 (38249.86)	0.9847 (2.5833)
aspirin 100	36	3567.58 (4163.36)	0.03085 (1.2562)	41	89952.9 (561954.34)	1.1856 (3.4496)
amoxicillin 500	51	5503.88 (31561.04)	4.0855 (6.7088)	156	1282.76 (2106.23)	2.5683 (4.6342)
aciclovir 200	30	2513.33 (3556.39)	0.1756 (0.3487)	29	9641.9 (30915.89)	0.7068 (1.9487)
diazepam 5	31	1464.19 (1333.9)	0.0281 (0.0170)	23	311.26 (489.03)	0.1424 (0.2413)
captopril 12,5	34	7159.74 (9238.61)	2.0732 (6.3786)	14	362.29 (773.69)	2.2564 (5.2408)
atenolol 50	18	1961.67 (1605.46)	0.0487 (0.0742)	13	102443.85 (243066.2)	0.1162 (0.1729)

The results from the regression model and the mixed effects model are shown on Tables 3 and 4 respectively. The confidence intervals of the time trends for Sao Paolo and Paraiba were estimated using Bootstrap [46]. Time trends were considered as statistically significant if the confidence intervals do not contain the value of 1. Complete results from both types of models, including point estimates and confidence intervals for all the coefficients can be found in the additional files (see Additional files 3 and 4: Tables S3 and S4).

**Table 3:** Regression Results for Paraiba (PB) and Sao Paulo (SP), showing time trend estimates and 95 % confidence intervals, adjusted R^2^ and p-value from the F test

Drug	Sample size	Time trend estimate PB	Time trend estimate SP	Adj R^2^	P-value
Aciclovir 200	59	0.878 (0.795, 0.981)	0.931 (0.783, 1.131)	0.373	<0.0001
Amoxicillin 500	207	1.100 (0.986, 1.268)	0.981 (0.916, 1.058)	0.695	<0.0001
Aspirin 100	77	1.032 (0.919, 1.243)	1.045 (0.925, 1.246)	0.644	<0.0001
Atenolol 50	31	0.939 (0.762, 1.076)	0.966 (0.694, 1.553)	0.09	0.171
Captopril 12,5	48	1.218 (0.998, 1.466)	1.053 (0.703, 1.541)	0.65	<0.0001
Diazepam 5	54	1.059 (0.986, 1.186)	0.999 (0.858, 1.178)	0.338	<0.0001
Dipyrone 500	269	1.070 (0.944, 1.260)	0.993 (0.936, 1.052)	0.481	<0.0001
Enalapril 10	42	1.050 (0.857, 1.388)	1.286 (0.950, 1.567)	0.227	0.008
Fluoxetin 20	36	0.931 (0.523, 1.477)	1.098 (0.826, 1.585)	0.085	0.151
Furosemide 40	84	1.049 (0.921, 1.167)	0.961 (0.789, 1.140)	0.575	<0.0001
Glibenclamide 5	41	1.074 (0.938, 1.235)	1.201 (0.851, 2.133)	0.22	0.011
Hydrochlorothiazide 25	30	1.237 (0.987, 2.255)	0.856 (0.489, 1.174)	0.371	0.003
Metformin 850	37	0.856 (0.777, 0.928)	0.939 (0.771, 1.037)	0.242	0.011
Omeprazol 20	111	0.862 (0.692, 1.046)	0.967 (0.860, 1.082)	0.368	<0.0001
Paracetamol 500	170	1.146 (1.031, 1.268)	1.074 (0.962, 1.190)	0.735	<0.0001
Propranolol 40	57	1.022 (0.860, 1.344)	1.024 (0.826, 1.295)	0.2	0.003
Ranitidine 150	105	1.034 (0.957, 1.115)	1.127 (1.021, 1.246)	0.478	<0.0001
Tramadol 50	55	0.761 (0.681, 0.949)	1.067 (0.847, 1.466)	0.46	<0.0001
Warfarin 5	40	0.837 (0.759, 0.975)	1.035 (0.899, 1.218)	0.626	<0.0001

**Table 4:** Mixed effects model results for paraiba and sao paulo. Time trends estimates and 95 % confidence intervals are shown

Drug	Time trend estimate PB	Time Trend estimate SP
Aciclovir 200	0.904 (0.845, 0.971)	1.055 (0.925, 1.213)
Amoxicillin 500	1.035 (0.926, 1.166)	1.003 (0.934, 1.074)
Aspirin 100	1.018 (0.871, 1.163)	1.021 (0.864, 1.199)
Atenolol 50	0.897 (0.787, 1.032)	1.000 (0.775, 1.281)
Captopril 12,5	1.258 (1.024, 1.554)	1.085 (0.736, 1.505)
Diazepam 5	1.031 (0.975, 1.097)	1.025 (0.948, 1.111)
Dipyrone 500	1.094 (0.966, 1.232)	0.977 (0.927, 1.033)
Enalapril 10	1.074 (0.942, 1.241)	1.239 (1.071, 1.437)
Fluoxetin 20	0.924 (0.688, 1.218)	1.095 (0.783, 1.467)
Furosemide 40	1.084 (0.956, 1.220)	0.939 (0.811, 1.079)
Glibenclamide 5	1.087 (0.946, 1.232)	1.064 (0.767, 1.454)
Hydrochlorothiazide 25	0.981 (0.895, 1.066)	1.140 (0.931, 1.385)
Metformin 850	0.860 (0.779, 0.944)	0.943 (0.820, 1.099)
Omeprazol 20	0.813 (0.656, 0.987)	1.004 (0.897, 1.132)
Paracetamol 500	1.151 (1.017, 1.296)	1.039 (0.943, 1.144)
Propranolol 40	1.000 (0.889, 1.115)	0.983 (0.840, 1.156)
Ranitidine 150	1.022 (0.934, 1.127)	1.053 (0.955, 1.161)
Tramadol 50	0.713 (0.615, 0.819)	0.922 (0.770, 1.106)
Warfarin 5	0.833 (0.772, 0.902)	1.002 (0.875, 1.152)

### a) Results from the primary analysis

The simple linear regression models revealed that in Paraiba the unit price of 5 of the 19 medicines investigated changed significantly with time. The unit price of four of those (Aciclovir, Metformin, Tramadol and Warfarin) decreased over time, while the unit price of Paracetamol increased over time. In São Paulo, no drug showed a significantly decreasing time trend, and the unit price of only Ranitidine showed a significant increasing trend.

Evidence of stationarity was found from the Augmented Dickey-Fuller test.

### b) Results from the secondary analysis

The more complex mixed effects model was used as secondary analysis and revealed that 8 out of the 19 medicines investigated changed significantly with time. Seven of the medicines with significant time trends were in Paraiba, with two of them showing an increasing trend (Captopril, Paracetamol) and five showing a decreasing trend (Aciclovir, Metformin, Omeprazol, Tramadol, and Warfarin). In the state of São Paulo only one medicine (Enalapril) showed a significant time trend, where its unit price increased over time.

## Discussion

### What does the database show with regards to changes in drug prices over time?

The findings from our analysis suggest that there is no pattern of consistent decreases of prices during the five-year period for which the prices were analyzed. The medicines that were reviewed in the analysis represented widely used and prescribed medicines, so the results are suggestive of the fact that the existence of the BPS tool has not lead to consistent purchase price decreases for medicines in Paraiba and São Paulo.

### What does the database show with regards to comparative drug pricing between two socioeconomically different Brazilian states?

The results of the simple linear time-trend regression analysis revealed that the unit price of only 5 out of the 19 medicines either significantly increased or significantly decreased every year in the state of Paraiba. Conversely, this analysis revealed a significant change over time of the unit price of only one medicine in the state of São Paulo. The results from the more complex mixed effects model indicated that 8 drugs are significantly changing over time, seven in the state of Paraiba and only one in the state of São Paulo. Both of these analyses revealed a similar medicine price pattern for Paraiba against very limited evidence for São Paulo.

Potential explanations for the observed difference in results between the two states cannot be explained through policies and regulations that regulate drug prices in Brazil. While each Brazilian state is responsible for the purchasing and procurement of medicines, as well as for the allocation of funds for the purchase of medicines, the Federal Government regulates price and payment practices in each state. All states are also under surveillance of the Regulation Chamber of Drug Market (CMED, *Câmara de Regulação do Mercado de Medicamentos*) [47], which regulates drug prices in the pharmaceutical industry [48]. We were unable to find any difference in drug policies between São Paulo and Paraiba that would have an effect on the observed differences in price patterns between the two states. However, there are other factors that were not considered during analysis, such as the local supply conditions of each state, inefficiencies in their health systems, embezzlement and fraud, as well as a lack of respect for laws that regulate drug prices that may explain the observed differences in results between Paraiba and São Paulo.

### What does this examination say about the BPS’ ability to work as a transparency measure to decrease drug prices?

The results from our two analyses showed evidence of unit price decrease over time for 5 out of the 19 drugs, all in the state of Paraiba, and none in the state of São Paulo. Our analysis therefore suggest that transparency portals such as the BPS may not be an effective tool for decreasing drug prices in those two states, particularly in São Paulo, and suggestively in Brazil as a whole. If we refer back to Schargrodsky *et al.’s* study [15] that analyzed a similar transparency measure for drug prices in Argentina, our findings are similar. Both studies suggests that any observed decrease in drug prices upon the initial implementation of the policy may have been caused by the anticipation of possible chances of detection and punishment if prices were well beyond the average. One major failing of the Argentinian initiative, which may also be the same for the BPS tool, was the lack of action from the government following the results of the survey. Evidently, inefficiencies and corrupt practices continued, which ultimately led to an eventual increase in drug prices. In Brazil, this is also indicative of the lack of regulation and action on the part of the Regulation Chamber of Drug Market (CMED) which is responsible for regulating drug prices in the pharmaceutical industry. It is clear that the BPS does not fully capture the theorized benefits of pricing transparency, and alone is not effective at driving down purchasing prices.

### Limitations

The most important limitation within our study is the sample size (number of purchase reports) for each drug that was used to analyze drug prices, for which some medicine pairings are not comparable in quantity. A larger sample size may have revealed more significant increases or decreases in medicine price in either São Paulo or Paraiba. Moreover, we do not have similar comparable purchase data without the presence of the portal, so we cannot know if the changes in unit price over time are due to the BPS or if they would have happened without this transparency tool.

Another limitation in our data lies in the existence of the Ministry of Health’s Popular Pharmacy Program, which was launched in 2004, during the study period. With this Program, citizens can purchase medicines on Brazil’s RENAME at a 90 % discount, which is subsidized by the government [49].The Ministry of Health supplies the program by manufacturing the medicines through a national public foundation [50]. Since the majority of the selected medicines in this study were also available for purchase at a discounted rate through the Popular Pharmacy Program [51] this measure may have an effect on drug prices based on the supply and demand present in the market. Perhaps future studies can investigate the medicines not present on the Popular Pharmacy list to limit external factors that could affect price. The type of medicine purchased by the institutions is another limitation because there are three types of medicines in the Brazilian market: originator brand, generic and similar [11]. The difference between a similar and generic drug is that the generic drug is bioequivalent to the original drug and has proven to have the same efficacy, safety and quality, whereas the similar drug is a copy of the reference, but there is no proof of bioequivalence [10]. Although the prices drawn from the BPS were those of generics, (which are known to be more expensive than similars), the BPS does not differentiate between generic and similar drugs, and thus this could affect the results of the regression model.

Finally, the BPS does not differentiate between the types of drug suppliers, which makes it difficult to conclude to what extent this affects our analysis. The type of supplier (manufacturer, distributer, or wholesaler, and local or international) has a direct effect on medicine price through different markups and it is important to take this information into consideration when conducting an analysis of price trends over time. Our data identified 82 % of suppliers to be wholesale distributers, but whether they were foreign-sourced or local was unclear.

## Conclusion

We found a unique interplay of the effects that transparency has had on restraining drug prices. Even after controlling for procurement quantities (economies of scale), supplier and institution/purchaser (potential sources of corruption), our analysis found that there is no consistent decrease in prices across all medicine groups. While the BPS has increased access to information on drug purchasing in Brazil, it has not shown to lead to consistent reductions in drug purchase prices for some of the most widely used medicines within a five-year time span. This is indicative of a limited model for addressing the challenges in pharmaceutical procurement. It is clear that Brazil’s BPS tool alone is a necessary but insufficient tool to address the risks of corruption, nor does it provide enough incentive to pharmaceutical suppliers to compete with lower prices.

Even though pricing transparency should allow for a decrease in prices, evidence reveals that additional measures must be taken to warrant such optimal price patterns. Comparison to other Mercosur countries with pricing transparency initiatives has shown that countries with additional measures embedded within transparency tools (such as competitive tendering) can effectively reduce prices. Of note are the countries with pricing transparency tools similar to Brazil (e.g. Argentina) that had not shown the same successes in lowering prices until they had instituted additional transparency measures and incentives [15, 27, 31]. Transparency measures such as BPS can be improved considerably if they include incentives and rewards for “good” behaviours and penalties for “bad” behaviours, as well as further attempts to increase price transparency and competition among suppliers.

### Opportunities for further research

Given the limitations of our study (such as the small number of drugs analyzed and the fact that our research was conducted in only 2 out of 27 Brazilian states) greater analysis that accounts for these and other factors should be explored in order to have more conclusive and robust results. Further research is needed to deeply analyse the extent to which the BPS has fulfilled its mandated objectives that have an effect on drug prices. Further exploration is needed in terms of understanding BPS’s effectiveness in managing market information of medicines and other health products, as well as the degree to which excluding non-federal institutions in the mandatory reporting of purchasing information affects the market information available in the BPS. Moreover, it is important to analyse the degree to which the BPS is used as a guideline for drug purchases throughout Brazil, particularly in low-resource settings where Internet might not be available. It may be illuminating to explore the positive and negative consequences of creating an institution solely dedicated to monitoring drug price trends and following up when irregularities are found that may cause unexcused price increases. These factors may not only conclude the extent to which the BPS helps decrease drug prices in Brazil, but may also help formulate recommendations for improving its success and to help improve better access to medicines throughout Brazil.

## Additional files

Additional file 1:
**Paraiba Descriptive Statistical Analysis.** This table provides the descriptive statistical analysis for the State of Paraiba. Additional file 2:
**Sao Paulo Descriptive Statistical Analysis.** This table provides the descriptive statistical analysis for the State of Sao Paulo. Additional file 3:
**Linear Regression Model Results.** This table provides the detailed results of the linear regression model analysis. Additional file 4:
**Mixed Effects Model Results.** This table provides the detailed results of the mixed effects model analysis.

## Abbreviations

EML: Essential medicines list
BPPH: Banco de preços praticados na área hospitalar
BPS: Banco de preços em saúde
CEASS: Central de abastecimiento y suministros
CENABAST: Central nacional de abastecimiento
CMED: Câmara de regulação do mercado de medicamentos
MERCOSUR: Mercado común del sur
MeTA: Medicines transparency alliance
NGO: Non-governmental organization
PNMEBOL: Programa de medicamentos esenciales de bolivia
RENAME: Relação nacional de medicamentos essenciais
SIASG: Sistema integrado de administração de serviços gerais
SICIAP: Sistema de información y control de inventario automatizado
SUS: Sistema unico de saude
UCAMAE: Unidad centralizada adquisiciones
